# ATR-FTIR spectroscopy and LDA: a rapid, non-destructive and cost effective strategy to trace the geographical origin of *Cannabis sativa* L.

**DOI:** 10.1093/fsr/owae016

**Published:** 2024-02-07

**Authors:** Mariana F Ramos, Chad A Kinney, João A Coblinski, Mauro S Fett, Deborah P Dick, Flávio A de Oliveira Camargo

**Affiliations:** Federal University of Rio Grande do Sul, Faculty of Agronomy, Department of Soil Science, Porto Alegre, Brazil; Colorado State University-Pueblo, Pueblo, CO, USA; Chemistry Department, Colorado State University-Pueblo, Pueblo, CO, USA; Federal University of Rio Grande do Sul, Faculty of Agronomy, Department of Soil Science, Porto Alegre, Brazil; Brazilian Federal Police, Porto Alegre, Brazil; Federal University of Rio Grande do Sul, Institute of Chemistry, Porto Alegre, Brazil; Federal University of Rio Grande do Sul, Faculty of Agronomy, Department of Soil Science, Porto Alegre, Brazil

**Keywords:** forensic sciences, infrared spectroscopy, marijuana, hemp, chemometrics

## Abstract

*Cannabis* recreational and/or medicinal use has been legalized in the past years in many states and countries. As a consequence, many *Cannabis* growers and product developers have emerged in a new market throughout the world; at the same time, issues regarding questionable quality control have also risen, as several reports on *Cannabis* users’ health-related problems caused by inaccurate labeling content in *Cannabis*-based medicines, edibles or other derivatives are being published and brought out to the public’s attention. These facts make traceability methodologies crucial whether for forensic use, such as drug trafficking eradication, or for quality control purposes of legal *Cannabis* and of products derived from it. Hence, the objective of this study was to analyze *Cannabis* by means of attenuated total reflectance Fourier transform infrared spectroscopy (ATR-FTIR) to assess the capability of this technique to trace the geographical origin of *Cannabis* cultivated in Brazil and in Colorado, USA. Forty-seven samples from Brazil and 18 samples from Colorado were analyzed by ATR-FTIR. Linear discriminant analysis (LDA) was employed to source the samples. The combination of ATR-FTIR and LDA achieved up to 95.23% accuracy in assigning *Cannabis* samples to their geographical locations of origin in Brazil and up to 100% in Colorado.

## Introduction

The number of *Cannabis* users was of 200 million in 2019 [1], and it is the most used illicit drug worldwide despite its use being illegal in several countries [2]. The combined amounts of *Cannabis* resin and herbal *Cannabis* accounted for over 744 300 kg seized in the European Union, Turkey, Norway and the UK in 2019 [3]. Moreover, there is an increasing tendency in the amounts of marijuana being seized in Brazil as well as in the USA. In Brazil, either the amount of marijuana or the combined amount of marijuana, hashish and “skunk” seized by the Brazilian Federal Police shows a tendency of growth since 1995, surpassing 350 000 kg in 2017, and even 410 200 kg in 2021 [4]. Data available on the amount of total bulk processed marijuana seized in the USA [5] also show a tendency of growth, although the time series is smaller, which makes data interpretation more complicated.

As to the medicinal and/or recreational use of marijuana, which has been legalized in many countries, several *Cannabis*-based products have become available in the market, as well as concerns regarding their safety [6–10]. Several patients have been admitted in hospitals due to issues caused by the use of adulterated [10–12] or synthetic marijuana [7]. Deaths occasioned by marijuana-induced complications such as that of a neonate with marijuana toxicity to her organs [13] or those of men who consumed marijuana-based edibles [14, 15].

Since the quantities available for *Cannabis* analysis are frequently quite small, and analysis time and inputs are important factors in most laboratories across the world, it is important to find instrumentation that meet these bottlenecks. Spectroscopic analyses such as attenuated total reflectance Fourier transform infrared spectroscopy (ATR-FTIR) are rapid, require only a few milligrams of sample, little or no sample preparation, and are non-destructive. For these reasons, ATR-FTIR is a valuable asset for the forensic sciences, being applied in the identification of asphalt fingerprints [16], discrimination of blood samples [17], analysis of bloodstains [18], and identification of claws for wildlife forensics [19]. Regarding *Cannabis*, ATR-FTIR has been employed to characterize the oil, seeds and flour of hemp [20], the applicability evaluation of *Cannabis* protein powder in the remediation of contaminated groundwater [21], as well as the assessment of hemp fibers [22].

Absorptions in the mid-infrared spectrum (4 000–400 cm^−1^) are related to fundamental vibrations within molecules. Vibrational and rotational changes in molecules occur in response to their interaction with infrared light, causing their chemical bonds to vibrate more energically [23]. Reflectance spectroscopy investigates chemical characteristics and behaviour of a given object by means of its interaction with electromagnetic energy [24]. When energy interacts with an object, part of it may be absorbed, transmitted and/or reflected. Regarding ATR-FTIR, the instrumentation emits an infrared beam that travels from a medium of high refractive index (such as zinc selenide crystal) to a medium of low refractive index, ie, the sample [25]. A part of the light is reflected back into the sample, and a fraction of the light escapes the high refractive index medium and extends beyond the surface in the form of waves, called evanescent waves, which interact with the sample, and the absorbance is translated into a spectrum [25].

As traceability is becoming increasingly important to protect consumers, honest producers [26] and assist police enforcement by accurately sourcing *Cannabis*, the aim of this study is to trace the geographical origin of *Cannabis* samples from Brazil and from the USA, according to their infrared spectra, determined by ATR-FTIR.

## Material and methods

### Cannabis samples

**Figure 1:**
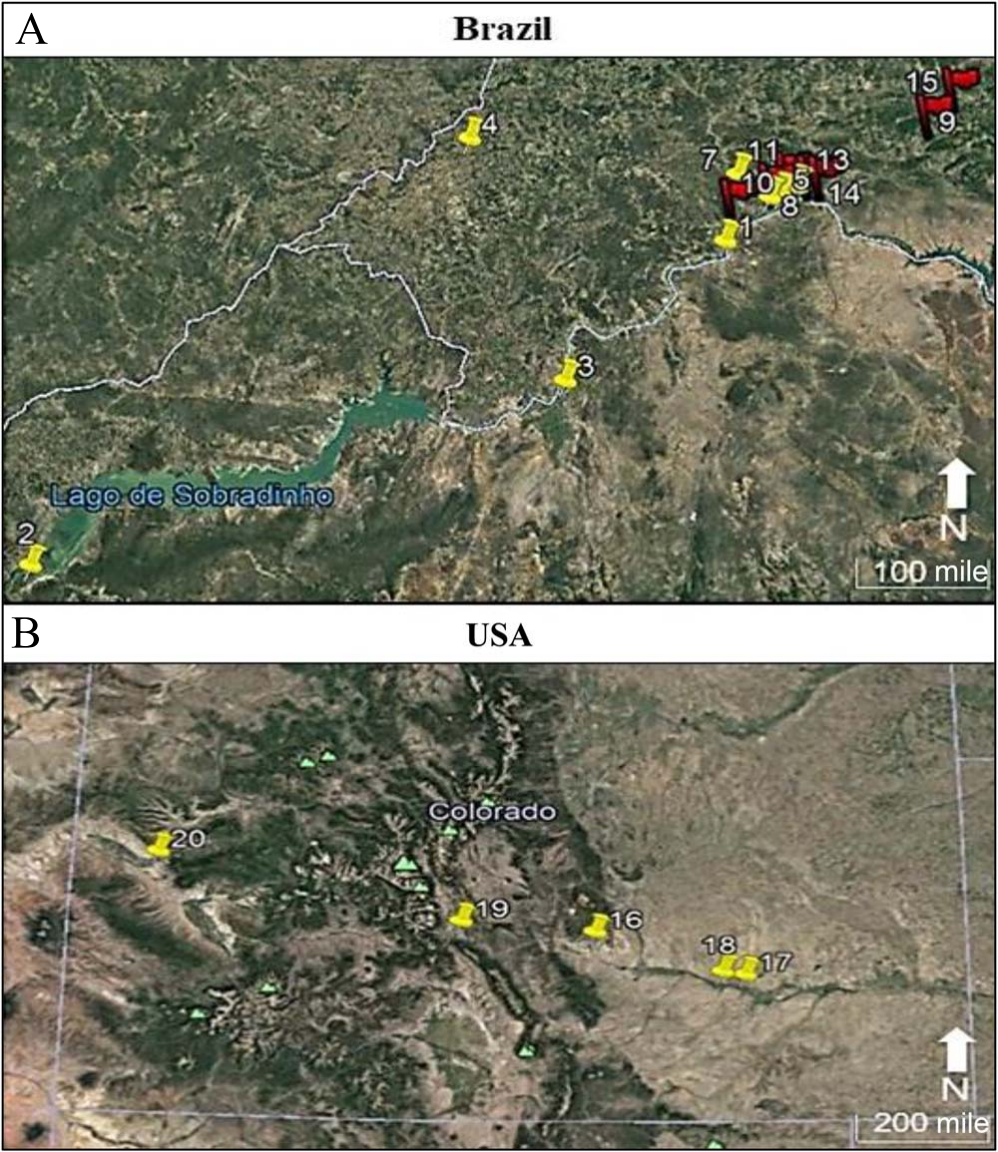
Sampling points in Brazil (A) and in Colorado (B). The red-flagged Brazilian sampling points mark the 2017 seizure operation locations, whereas the yellow sampling points represent the 2014 operation locations. The Brazilian sampling points number 6 and 12 do not appear in this Figure for they were visually overlapped by the majority of sampling point. mi: mile.

The Brazilian samples are originated from two seizure operations performed by the Brazilian Federal Police in 2014 and in 2017 (Figure 1A). In the first operation 26 *Cannabis* samples (entire plant, from aerial parts to roots) were collected from eight sampling points comprising the northeastern states of Pernambuco and Bahia, and in the second operation 21 plants were collected from seven sampling points (Figure 1A). At least two neighbouring samples per sampling point were collected.

The samples from the 2014 operation had already been the subject of a previous study (data not shown), being oven-dried at 105°C (221°F) for 24 h, whereas the samples from 2017 had not been part of any study and were oven-dried at 40°C (104°F) for 7 d. All samples were ground using a mortar and pestle. The samples were kept at room temperature and protected from light. Refrigeration was not possible due to legal issues.

The samples from Colorado, a western state of the USA, are from five hemp-producing farms (Figure 1B). Twelve samples (aerial parts) were acquired. Six samples were split in half in order to conduct a temperature test, resulting in the pairing samples numbered 174 and 175, 182 and 183, 184 and 185, 186 and 187, and 188 and 189. It was not possible to do so with all the samples due to their small quantities. One half of these six samples was air-dried for 7 d at room temperature and protected from light, while the other half was oven-dried at 40°C (104°F) for 7 d as were the Brazilian samples from 2017. After the drying step, all samples were ground using mortar and pestle, put into falcon tubes and kept refrigerated at 7°C (44.6°F).

Concerning the distance between samples, the farthest Brazilian sampling points/codes (2 and 9) are 447.7 km (~278.2 mile) apart, and the closest ones (5 and 11) are 0.8 km (~0.5 mile) from each other; as to the samples from Colorado, the sampling points 17 and 20 are the most distant ones, being 410.6 km (~255.13 mile) apart, while the sampling points 17 and 18 are 15.3 km (~9.51 mile) away from each other.

### Spectroscopic analysis

ATR-FTIR analyses of the samples from Brazil were performed in a Nicolet-6700 Thermo Scientific (Madison, WI, USA), with germanium crystal, in the spectral range between 4 000 and 500 cm^−1^, resolution of 4 cm^−1^, 32 scans per spectrum and one replicate. A blank, consisting of an analysis with no samples, was run between samples. The analyses of the samples from Colorado were performed in triplicates using a Nicolet™ iS™50 Thermo Scientific spectrometer (Madison, WI, USA). All samples from Colorado were analyzed using three approaches changing the number of scans and resolution: (i) 32 scans, resolution 4 cm^−1^, (ii) 100 scans, resolution 4 cm^−1^, and (iii) 100 scans, resolution 2 cm^−1^. The wavenumber range (4 000 to 400 cm^−1^) remained the same in all analyses. Triplicates were averaged using the ChemoStat software (Madison, WI, USA). Spectra from Colorado were baseline corrected using the instrumentation software. A blank was run every one hour, according to the instrumentation settings.

### Statistical analyses

Linear discriminant analysis (LDA) was carried out using the MASS package [27] in R software [28]. Spectra obtained through ATR-FTIR were analyzed by the LDA in its entirety; all wavenumbers were evaluated as a whole. The samples that did not have known geographical origin were excluded from the LDA (Table 1).

**Table 1:** Codes and groups of samples from Brazil and the USA employed in the linear discriminant analysis (LDA).

**Brazil**								**USA**			
**2014**				**2017**							
**S**	**C**	**G**	**V**	**S**	**C**	**G**	**V**	**S**	**C**	**G**	**V**
94	1	A	-	151	9	A	-	174^od^	16	A	T1
95	1	A	-	152	9	A	-	175^od^	16	A	T1
96	1	A	-	153	9	A	-	176^nd^	16	A	T1
97	-	-	-	-	-	-	-	177^nd^	16	A	T1
98^*^	-	-	-	154	10	B	-	182^od^	17	B	Cherry Wine
99	2	B	-	155	10	B	-	183^nd^	17	B	Cherry Wine
100	2	B	-	156	10	B	-	184^od^	17	B	Cherry Wine
101	2	B	-	157	11	C	-	185^nd^	17	B	Cherry Wine
100	-	-	-	158	11	C	-	186^od^	17	B	Cherry Wine
101	-	-	-	159	11	C	-	187^nd^	17	B	Cherry Wine
104	3	C	-	160	12	D	-	188^od^	18	C	Cherry Wine
105	3	C	-	161	12	D	-	189^nd^	18	C	Cherry Wine
106	4	D	-	162	12	D	-	193^nd^	19	D	-
107	4	D	-	163	13	E	-	194^nd^	19	D	-
108	-	-	-	164	13	E	-	195^nd^	19	D	-
109	-	-	-	165	13	E	-	199^nd^	20	E	-
110	5	E	-	166	14	F	-	200^nd^	20	E	-
111	5	E	-	167	14	F	-	201^nd^	20	E	-
112	5	E	-	168	14	F	-				
113	5	E	-	169	15	G	-				
114	5	E	-	170	15	G	-				
115	5	E	-	171	15	G	-				
116	6	F	-								
117	6	F	-								
118	6	F	-								
119	7	G	-								
120	-	-	-								
121	7	G	-								
122	8	H	-								
123	8	H	-								
124	8	H	-								
Extra 3^*^	-	-	-								

S: sample code; G: LDA group; C: sampling code; V: plant variety.

* Samples excluded from the LDA due to unknown geographical origin.

^od^: oven-dried sample;

^nd^: natural-dried sample.

*T*-test for independent samples, by groups, with 5% significance was performed using the Statistica software (TIBCO Software Inc., Palo Alto, CA, USA), aiming to verify if the Brazilian samples from 2014 and from 2017 were statistically different, since they not only had different ages but they had been dried differently. The air-dried and oven-dried samples from Colorado underwent the same analysis.

To perform the LDA it was necessary to assign codes to the samples: those from the same geographical origin formed one group and this group received a code (Table 1).

In the LDAs where data from both 2014 and 2017 were compared together, in the same dataset, the alphabetical codes assigned to the 2017 samples started from the letter “H” (which was the last letter code for the 2014 samples) onwards, e.g., the samples that formed the first group of 2017 were called group “I”, the next one group “J” and so on.

In order to make the analysis approach of the samples from Colorado as similar to that of the Brazilian samples, we performed three tests that were also analyzed by the LDA and the *t*-test, on the spectra collected from the American samples, as follows: (i) reduction in the spectral range, from 4 000–400 to 4 000–500 cm^−1^ (this reduction was maintained in the other tests as well), (ii) only the first replicate was analyzed, that is, the second and third spectroscopic recordings of the Colorado samples were excluded in this approach, and (iii) spectra without baseline correction. To simplify, the approaches with triplicates, baseline correction, and wavenumber range of 4 000–400 cm^−1^ will be referred to as “regular”, and the other approaches will be identified as “modified”.

Other analyses such as principal components analysis, hierarchical cluster analysis, and k-means clustering were also tested separately but were not able to accurately cluster the samples, causing these results to be discarded.

## Results and discussion

**Figure 2:**
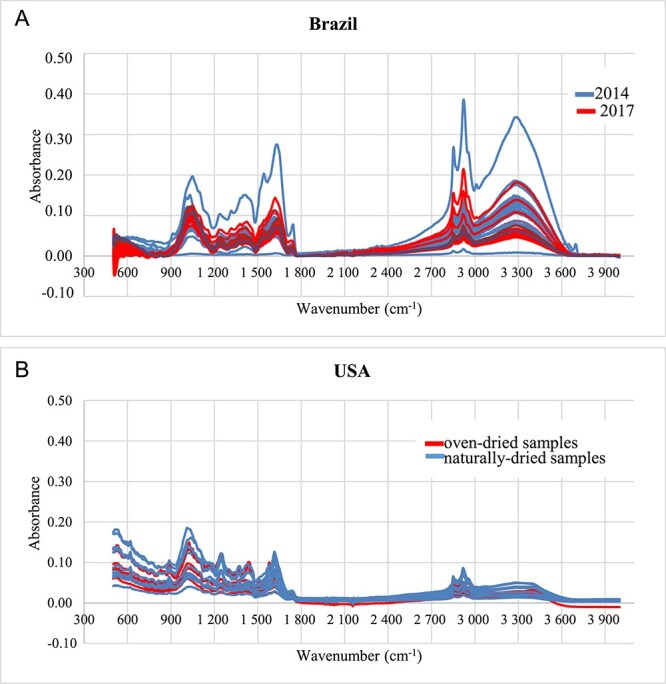
Spectra of the Brazilian (A) and American (B) samples. Data regarding the analyses with 32 scans, resolution of 4 cm^−1^ and spectral range of 4 000–500 cm^−1^.

All spectra were plotted with the same parameters, ie, the same spectral range, no baseline correction, same number of scans and resolution (Figure 2). Some spectra from the 2014 seizure operation present very distinct absorbance intensities, especially in the spectral range from 3 700 through 2 900 cm^−1^, which may be generally associated with X–H stretching bonds [23], and 1 800 through 900 cm^−1^, which partially comprises double bonds and the fingerprint region [23]. The samples from the 2017 operation are more similar to each other.

The attributions to the main functional groups present in all of the samples are listed in Table 2.

**Table 2:** Attributions to the main functional groups detected in the samples from Brazil and Colorado [29]

**Bands (cm** ^**−1**^ **)**	**Attributions**	**Functional groups**
3 279	O-H stretching in H-bonds	All kinds of organic compounds that possess OH, except metal-OH bonds which usually occur at higher wavenumbers (3 600 cm^−1^+)
2 917	C-H axial stretching	R-CH_3_
2 848	C-H axial stretching	R-CH_2_-R or tertiary -C-H
1 733	C=O stretching of -COOH and/or ketones	
1 245	C-O bending of -COOH	
1 144	C-O bending of alcohols or aliphatic ethers	
~1 100	C-O-C bending, C-O stretching of polysaccharides	Either carbohydrates, esters, carboxylic acids
1 014	C-O bending and /or Si-O	Either carbohydrates, esters, carboxylic acids

### Statistical analyses of the samples from Brazil

There is statistical difference, at 5% significance, between the 2014 and 2017 samples from Brazil regarding the wavenumbers from 903.93 cm^−1^ to 674.41 cm^−1^, 993.19–987.53 cm^−1^, 1 221.30–1 149.04 cm^−1^, 1 362.99–1 270.89 cm^−1^, 1 448.00–1 378.57 cm^−1^, 1 507.50–1 452.25 cm^−1^ (all of these comprising the fingerprint region [23]). This statistical difference was also observed for the same samples between the wavenumbers 1 612.35 and 1 527.34 cm^−1^, 1 721.44–1 690.27 cm^−1^, 1 785.20–1 763.95 cm^−1^, 1 795.12–1 792.29 cm^−1^, which are all part of the double-bond region; and finally, from 3 685.17 cm^−1^ to 1 807.87 cm^−1^, and from 3 982.71 cm^−1^ to 3 703.59 cm^−1^, which are all part of the X–H stretching region [23]. Samples from the same geographical origin were collected as closely to each other as possible in order to diminish the variability among them, such as that caused by different soil types [30, 31], landscapes and land reliefs [32, 33] as well as microclimates [34, 35].

The LDA accuracy for samples from the 2014 seizure operation is of 62.50% and for those from the 2017 operation is of 95.23%. It is assumed that the misclassified samples are from the same variety, which would occasion similar chemical and physical structures that, being detected in the spectroscopic analysis, could make them be clustered together in the LDA. In total, nine out of the 26 samples from 2014 were misclassified, leading to a reduced LDA accuracy.

When analysing the 2014 and 2017 samples together as only one dataset, the LDA accuracy is of 88.88%. Samples from all groups of the 2014 seizure were misclassified as belonging to the group E, except the samples from groups D and H; only group D had all its samples correctly classified (Table 3). Regarding the samples from the 2017 seizure operation, only one sample from group D was misclassified, being assigned to group C (Table 4). Although the 2017 samples are from relatively close geographical locations (Figure 1A), when compared to the distances between the 2014 samples, the LDA was able to accurately assign 95.23% of them.

**Table 3:** Distribution matrix of the Brazilian samples (2014) assigned by linear discriminant analysis (LDA) regarding their geographical origin.

**Samples from the 2014 seizure operation**								
	**A**	**B**	**C**	**D**	**E**	**F**	**G**	**H**
**A**	2	0	0	0	0	0	0	0
**B**	0	1	0	0	1	0	0	0
**C**	0	0	1	0	0	0	0	0
**D**	0	0	0	2	0	0	0	0
**E**	1	1	1	0	5	2	1	0
**F**	0	0	0	0	0	1	0	1
**G**	0	0	0	0	0	0	1	0
**H**	0	1	0	0	0	0	0	2

**Table 4:** Distribution matrix of the Brazilian samples (2017) assigned by linear discriminant analysis (LDA) regarding their geographical origin.

**Samples from the 2017 seizure operation**								
	**A**	**B**	**C**	**D**	**E**	**F**	**G**	**-**
**A**	3	0	0	0	0	0	0	-
**B**	0	3	0	0	0	0	0	-
**C**	0	0	3	1	0	0	0	-
**D**	0	0	0	2	0	0	0	-
**E**	0	0	0	0	3	0	0	-
**F**	0	0	0	0	0	3	0	-
**G**	0	0	0	0	0	0	3	-

The sum of the two first linear discriminants (LDs), which represents the majority of data variability, of the 2014 samples was of 52.85%, and that of the 2017 samples was of 80.77%. When all the samples were analyzed together, the sum of the LD1 and LD2 explains 84.19% of data variance.

When the samples from both seizure operations (2014 and 2017) were analyzed together (Table 5), as one dataset, all the samples from 2014 were correctly classified, contrarily to those from 2017, whose group I was the only one correctly classified. All other groups from 2017 had at least one sample misclassified.

**Table 5:** Distribution matrix of the Brazilian samples from 2014 and from 2017 combined, assigned by linear discriminant analysis (LDA) regarding their geographical origin.

	**A**	**B**	**C**	**D**	**E**	**F**	**G**	**H**	**I**	**J**	**K**	**L**	**M**	**N**	**O**
**A**	3	0	0	0	0	0	0	0	0	0	0	0	0	0	0
**B**	0	3	0	0	0	0	0	0	0	0	0	0	0	0	0
**C**	0	0	2	0	0	0	0	0	0	0	0	0	0	0	0
**D**	0	0	0	2	0	0	0	0	0	0	0	0	0	0	0
**E**	0	0	0	0	6	0	0	0	0	0	0	0	0	0	0
**F**	0	0	0	0	0	3	0	0	0	0	0	0	0	0	0
**G**	0	0	0	0	0	0	2	0	0	0	0	0	0	0	0
**H**	0	0	0	0	0	0	0	3	0	0	0	0	0	0	0
**I**	0	0	0	0	0	0	0	0	3	0	0	0	0	0	0
**J**	0	0	0	0	0	0	0	0	0	2	0	0	0	0	0
**K**	0	0	0	0	0	0	0	0	0	3	1	1	0	0	0
**L**	0	0	0	0	0	0	0	0	0	0	0	2	0	0	1
**M**	0	0	0	0	0	0	0	0	0	1	0	0	2	0	0
**N**	0	0	0	0	0	0	0	0	0	0	0	0	0	2	0
**O**	0	0	0	0	0	0	0	0	0	0	0	0	0	1	2

### Statistical analyses of the samples from the USA

#### “Regular” method—triplicates, baseline correction, and wavenumber range of 4 000–400 cm^−1^

Regarding the samples from Colorado, the *t*-test showed that the air-dried and oven-dried samples analyzed by ATR-FTIR with 32 scans and resolution of 4 cm^−1^ differ statistically, at 5% significance, in the following wavenumbers: 3 997.77 cm^−1^, 3 993.92–3 986.20 cm^−1^, 1 861.00–1 832.07 cm^−1^, 1 828.21–1 797.36 cm^−1^, 1 789.64–1 785.79 cm^−1^, 798.39–781.04 cm^−1^; the samples with spectra resulting from the analysis with 100 scans and resolution of 2 cm^−1^ differ in the wavenumbers 2 159.91 cm^−1^, 2 153.16–2 152.20 cm^−1^, 2 137.74–2 136.77 cm^−1^, 805.14–804.18 cm^−1^, 792.61–789.72 cm^−1^, 763.68–753.07 cm^−1^, whereas those analyzed with 100 scans and resolution of 4 cm^−1^ differ in 3 997.77–3 991.99 cm^−1^, 3 988.13–3 984.27 cm^−1^, 1 857.14–1 849.43 cm^−1^, 1 839.78–1 832.07 cm^−1^, 1 822.43–1 803.14 cm^−1^, 1 797.36 cm^−1^, 1 787.71–1 785.79 cm^−1^, and 794.54–784.89 cm^−1^.

The LDAs (Table 6) performed with the data from the analysis with 32 scans, resolution of 4 cm^−1^, with 100 scans, resolution of 4 cm^−1^, and with 100 scans, resolution of 2 cm^−1^, presented accuracies of 94.44%, 94.44%, and 100%, respectively. These results are similar to those of the Brazilian samples seized in 2017, but not to those from 2014, what indicates that factors responsible for plant tissue degradation, such as temperature and age, were determinant for the LDA accuracy. The particle size, considered an important factor in ATR analyses [36, 37], of all samples both from Brazil and from the United States was very similar visually, and therefore, this factor may be discarded from the discussion. Resolution was, apparently, the variable that most contributed to the LDA’s performance, being a key factor in *Cannabis* traceability by means of ATR.

**Table 6:** Distribution matrix of the American samples analyzed by the “regular” method, assigned by linear discriminant analysis (LDA) regarding their geographical origin.

**32 scans resolution 4 cm** ^**−1**^					
	**A**	**B**	**C**	**D**	**E**
**A**	3	0	0	0	0
**B**	0	6	0	0	0
**C**	0	0	2	0	0
**D**	1	0	0	3	0
**E**	0	0	0	0	3
**100 scans resolution 4 cm** ^**−1**^					
	**A**	**B**	**C**	**D**	**E**
**A**	4	0	0	0	0
**B**	0	6	1	0	0
**C**	0	0	1	0	0
**D**	0	0	0	3	0
**E**	0	0	0	0	3
**100 scans resolution 2 cm** ^**−1**^					
	**A**	**B**	**C**	**D**	**E**
**A**	4	0	0	0	0
**B**	0	6	0	0	0
**C**	0	0	2	0	0
**D**	0	0	0	3	0
**E**	0	0	0	0	3

The LDA shows that groups B, D and E had all their samples correctly classified in all approaches tested in the ATR analyses; only one sample of the group A was misclassified in the 32 scans approach. One sample of the group C was also misclassified in the 100 scans and resolution of 4 cm^−1^ approach. The sum of the first two linear discriminants (LD1 and LD2) accounts for 89.53% of data variance for the 32 scans and resolution of 4 cm^−1^, 82.34% for the 100 scans and resolution of 4 cm^−1^ and 90.14% for the 100 scans and resolution of 2 cm^−1^ analyses.

##### “Modified” methods Method 1 -- reduction in the spectral range, from 4 000–400 to 4 000–500 cm^−1^; triplicates, and baseline correction

The *t*-test showed that the following wavenumbers are significantly different, at 5% significance, in the 32 scans analysis: 3 997.77 cm^−1^, 3 993.92–3 986.20 cm^−1^, 1 861.00–1 832.07 cm^−1^, 1 828.21–1 797.36 cm^−1^, 1 789.64–1 785.79 cm^−1^, and 798.39–781.04 cm^−1^. As for the 100 scans resolution of 4 cm^−1^, the wavenumbers 3 997.77–3 991.99 cm^−1^, 3 988.13–3 984.27 cm^−1^, 1 857.14–1 849.43 cm^−1^, 1 839.78–1 832.07 cm^−1^, 1 822.43–1 803.14 cm^−1^, 1 797.36 cm^−1^, 1 787.71 cm^−1^, and 1 785.79 cm^−1^, 794.54–784.89 cm^−1^ differ between the oven-dried and air-dried samples, whereas the wavenumbers 2 159.91 cm^−1^, 2 153.16 cm^−1^ and 2 152.20 cm^−1^, 2 137.74 and 2 136.77 cm^−1^, 805.14 cm^−1^ and 804.18 cm^−1^, 792.61–789.72 cm^−1^, 763.68–753.07 cm^−1^ make the 100 scans resolution of 2 cm^−1^ analysis statistically different, concerning the drying method.

Regarding the LDA, the 32 scans analysis as well as the 100 scans and resolution of 4 cm^−1^ analysis showed 94.44% accuracy, whereas the 100 scans and resolution of 2 cm^−1^ was 100% accurate (Table 7).

**Table 7:** Distribution matrix of the American samples analyzed by the “method 1”, assigned by linear discriminant analysis (LDA) regarding their geographical origin.

**32 scans resolution 4 cm** ^**−1**^					
	**A**	**B**	**C**	**D**	**E**
**A**	3	0	0	0	0
**B**	0	6	0	0	0
**C**	0	0	2	0	0
**D**	1	0	0	3	0
**E**	0	0	0	0	3
**100 scans resolution 4 cm** ^**−1**^					
	**A**	**B**	**C**	**D**	**E**
**A**	3	0	0	0	0
**B**	0	6	0	0	0
**C**	0	0	2	0	0
**D**	1	0	0	3	0
**E**	0	0	0	0	3
**100 scans resolution 2 cm** ^**−1**^					
	**A**	**B**	**C**	**D**	**E**
**A**	4	0	0	0	0
**B**	0	6	0	0	0
**C**	0	0	2	0	0
**D**	0	0	0	3	0
**E**	0	0	0	0	3

The sum of the first two linear discriminants (LD1 and LD2) accounts for 89.57% of data variability in the 32 scans and resolution of 4 cm^−1^, 87.77% for the 100 scans and resolution of 4 cm^−1^ and 87.63% for the 100 scans and resolution of 2 cm^−1^ analyses.

##### Method 2—reduction in the spectral range, from 4 000–400 to 4 000–500 cm^−1^; first replicate analyzed, with baseline correction

When the *t*-test was performed using the samples from Colorado in the spectral range between 4 000–500 cm^−1^ and only considering the first reading, i.e., the first replicate of each sample, the oven-dried and air-dried samples differ from each other between 790.68 cm^−1^ and 784.89 cm^−1^ when analyzed with 32 scans, whereas in the analysis with 100 scans and resolution of 4 cm^−1^ the samples differ in the spectral ranges between 790.68–786.82 cm^−1^, 806.11 and 804.13 cm^−1^, and 831.18–827.32 cm^−1^, and in the analysis with 100 scans and resolution of 2 cm^−1^ the samples differ in the spectral ranges between 799.36 cm^−1^ and 792.61 cm^−1^.

Therefore, it is noticeable that the wavenumbers responsible for differing the samples regarding the drying method lie between a narrower range when the aforementioned parameters change, concentrating in the 780–830 cm^−1^ spectral range, which is part of the fingerprint region [23] of the infrared spectrum. Moreover, when the analysis performed with 32 scans, resolution of 4 cm^−1^, spectral range between 4 000–500 cm^−1^ without the baseline correction, the oven-dried and the air-dried samples did not show statistical differences.

The LDA showed that both the 32 scans and resolution of 4 cm^−1^ and the 100 scans and resolution of 2 cm^−1^ analyses achieved 100% accuracy, whereas the 100 scans and resolution of 4 cm^−1^ had 94.44% accuracy (Table 8). The sum of the LD1 and LD2 resulted in 79.69%, 78.60% and 89.19% of data variability, respectively.

**Table 8:** Distribution matrix of the American samples analyzed by the “method 2”, assigned by linear discriminant analysis (LDA) regarding their geographical origin.

**32 scans resolution 4 cm** ^**−1**^					
	**A**	**B**	**C**	**D**	**E**
**A**	4	0	0	0	0
**B**	0	6	0	0	0
**C**	0	0	2	0	0
**D**	0	0	0	3	0
**E**	0	0	0	0	3
**100 scans resolution 4 cm** ^**−1**^					
	**A**	**B**	**C**	**D**	**E**
**A**	4	0	0	0	0
**B**	0	6	1	0	0
**C**	0	0	1	0	0
**D**	0	0	0	3	0
**E**	0	0	0	0	3
**100 scans resolution 2 cm** ^**−1**^					
	**A**	**B**	**C**	**D**	**E**
**A**	4	0	0	0	0
**B**	0	6	0	0	0
**C**	0	0	2	0	0
**D**	0	0	0	3	0
**E**	0	0	0	0	3

##### Method 3—reduction in the spectral range, from 4 000–400 to 4 000–500 cm^−1^; first replicate and no baseline correction

When the *t*-test was performed in the samples from Colorado in the spectral range between 4 000–500 cm^−1^ and only considering the first reading, i.e., the first replicate of each sample, and without the baseline correction, i.e., the oven-dried and air-dried samples did not differ from each other when analyzed with 32 scans, whereas in the analysis with 100 scans and resolution of 4 cm^−1^ the samples differ in the spectral ranges 1 783.86–1 785.79 cm^−1^, 1 793.50 cm^−1^, 1 803.14–1 818.57 cm^−1^, 1 822.43–1 849.43 cm^−1^, 1 855.21–1 870.64 cm^−1^, 1 880.28–1 882.21 cm^−1^, 1 899.57–1 903.42 cm^−1^, 1 909.21–1 911.14 cm^−1^, 2 375.91–2 377.83 cm^−1^, 2 399.05 cm^−1^, 2 485.83–2 607.33 cm^−1^, and in the analysis with 100 scans and resolution of 2 cm^−1^ the samples differ in the spectral ranges 2 011.42–2 012.38 cm^−1^, 2 015.28 and 2 016.24 cm^−1^, 2 021.06 cm^−1^, 2 029.74 cm^−1^, 2 157.98 cm^−1^. When the parameters change, it caused the wavenumbers responsible for differing the samples regarding the drying method to lie in the 1 780–2 600 cm^−1^ spectral range, which is not part of the fingerprint region [23]. Additionally, as seen from the analysis performed with 32 scans, resolution of 4 cm^−1^, spectral range between 4 000 and 500 cm^−1^, changing the parameters strongly affected the *t*-test results.

Regarding the LDA, only group C had all their samples correctly classified in all approaches tested. Group E had just one sample misclassified in the approach with 100 scans and resolution of 4 cm^−1^ (Table 9). The accuracy for both the 32 scans analysis and the 100 scans and resolution of 2 cm^−1^ was of 88.89%, whereas for the 100 scans and resolution of 4 cm^−1^ it was slightly lower, 83.33%.

**Table 9:** Distribution matrix of the American samples analyzed by the “method 3” assigned by linear discriminant analysis (LDA) regarding their geographical origin.

**32 scans resolution 4 cm** ^**−1**^					
	**A**	**B**	**C**	**D**	**E**
**A**	4	1	0	1	0
**B**	0	5	0	0	0
**C**	0	0	2	0	0
**D**	0	0	0	2	0
**E**	0	0	0	0	3
**100 scans resolution 4 cm** ^**−1**^					
	**A**	**B**	**C**	**D**	**E**
**A**	3	1	0	0	0
**B**	1	5	0	0	0
**C**	0	0	2	0	0
**D**	0	0	0	3	1
**E**	0	0	0	0	2
**100 scans resolution 2 cm** ^**−1**^					
	**A**	**B**	**C**	**D**	**E**
**A**	3	0	0	1	0
**B**	0	6	0	0	0
**C**	0	0	2	0	0
**D**	1	0	0	2	0
**E**	0	0	0	0	3

The LDA performed with the ATR-FTIR “regular” methodologies consisting of 32 scans and resolution of 4 cm^−1^, 100 scans and resolution of 2 cm^−1^, and 100 scans and resolution of 4 cm^−1^ presented accuracies of 94.44%, 100% and 94.44%, respectively. These results are similar to those of the Brazilian samples seized in the 2017 police operation, but not to those seized in 2014. As mentioned before, the particle size of all the samples from Brazil and from the United States was visually identical; therefore, this factor can be excluded as a possible explanation to the results from the “regular” methodology. Similar plant age and temperature treatment seem to have an impact on the chemical and/or physical aspects of the samples, and consequently on the data. However, when the samples were assessed by the “modified” approach, the accuracy results were considerably lower for the 32 scans and the 100 scans resolution of 4 cm^−1^ approaches, being 88.88% for the first and 77.77% for the latter, but remained the same for the 100 scans resolution of 2 cm^−1^. These results indicate that doing replicates [38] and baseline correction improve accuracy and should be employed whenever possible.

## Conclusions

The combination of attenuated total reflectance spectroscopy, a rapid, non-destructive, cost effective analysis, and linear discriminant analysis, proved suitable to trace the geographical origin of *Cannabis sativa* L., with accuracies between 62.50% and 95.23% for the Brazilian samples and 94.44% and 100% for the samples from Colorado. Sample degradation caused by temperature and age played a major role in diminishing the traceability accuracy, as was observed in the samples from the 2014 seizure operation.

The quantity of replicates and the spectral range were decisive to the accuracy of the LDAs performed with the USA data, whereas the analyses with 32 scans and resolution of 4 cm^−1^ and with 100 scans and resolution of 4 cm^−1^ showed lower accuracies when only the first replicate was taken into consideration and the wavelength was shortened. Baseline correction also influenced the results and should be employed.

Further studies ought to be conducted with a larger sample set, different geographical locations and plant varieties in order to confirm the suitability of the present approach in the traceability of *Cannabis*.
